# A five-year quasi-experimental study to evaluate the impact of empiric antibiotic order sets on antibiotic use metrics among hospitalized adult patients

**DOI:** 10.1017/ice.2023.293

**Published:** 2024-05

**Authors:** Wesley D. Kufel, Jeffrey M. Steele, Rahul Mahapatra, Mitchell V. Brodey, Dongliang Wang, Kristopher M. Paolino, Paul Suits, Derek W. Empey, Stephen J. Thomas

**Affiliations:** 1 Binghamton University School of Pharmacy and Pharmaceutical Sciences, Binghamton, New York; 2 State University of New York Upstate Medical University, Syracuse, New York; 3 State University of New York Upstate University Hospital, Syracuse, New York

## Abstract

**Objective:**

Evaluation of adult antibiotic order sets (AOSs) on antibiotic stewardship metrics has been limited. The primary outcome was to evaluate the standardized antimicrobial administration ratio (SAAR). Secondary outcomes included antibiotic days of therapy (DOT) per 1,000 patient days (PD); selected antibiotic use; AOS utilization; *Clostridioides difficile* infection (CDI) cases; and clinicians’ perceptions of the AOS via a survey following the final study phase.

**Design:**

This 5-year, single-center, quasi-experimental study comprised 5 phases from 2017 to 2022 over 10-month periods between August 1 and May 31.

**Setting:**

The study was conducted in a 752-bed tertiary care, academic medical center.

**Intervention:**

Our institution implemented AOSs in the electronic medical record (EMR) for common infections among hospitalized adults.

**Results:**

For the primary outcome, a statistically significant decreases in SAAR were detected from phase 1 to phase 5 (1.0 vs 0.90; *P* < .001). A statistically significant decreases were detected in DOT per 1,000 PD (4,884 vs 3,939; *P* = .001), fluoroquinolone orders (407 vs 175; *P* < .001), carbapenem orders (147 vs 106; *P* = .024), and clindamycin orders (113 vs 73; *P* = .01). No statistically significant change in mean vancomycin orders was detected (991 vs 902; *P* = .221). A statistically significant decrease in CDI cases was also detected (7.8, vs 2.4; *P* = .002) but may have been attributable to changes in CDI case diagnosis. Clinicians indicated that the AOSs were easy to use overall and that they helped them select the appropriate antibiotics.

**Conclusions:**

Implementing AOS into the EMR was associated with a statistically significant reduction in SAAR, antibiotic DOT per 1,000 PD, selected antibiotic orders, and CDI cases.

Antibiotics are often prescribed for inappropriate indications and excessive durations.^1,2^ Inappropriate antibiotic use can contribute to the development of antibiotic resistance, which is a well-recognized public health threat with a substantial impact.^3,4^ One-third of hospital antibiotic orders involve potential problems such as prescribing an antibiotic without proper diagnostic testing or evaluation, prescribing an antibiotic unnecessarily, or prescribing excessive antibiotic durations for common infections.^5^ Thus, antibiotic stewardship initiatives are essential to improve antibiotic prescribing in the inpatient setting.^1,6^

The Centers for Disease Control and Prevention (CDC) Core Elements for Hospital Antibiotic Stewardship Programs suggest implementing antibiotic order sets (AOSs) to help improve antibiotic use.^5^ Order sets have been shown to improve the management of various disease states by promoting evidence-based care, thereby reducing inappropriate prescribing. However, most of these studies are limited to emergency departments, outpatient settings, or sepsis-specific protocols.^7–16^ Furthermore, many order sets have used clinical pathways that had to be accessed outside the electronic medical record (EMR), which may have deterred providers from readily accessing these resources. Incorporation of AOSs into the EMR may facilitate their use.

After the development and implementation of AOSs for common infections into our EMR, we evaluated antibiotic stewardship metrics including the standardized antimicrobial administration ratio (SAAR), antibiotic days of therapy (DOT) per 1,000 patient days (PD), use of targeted antibiotics, and *Clostridioides difficile* infection (CDI) cases. We also assessed clinician perceptions of the AOSs.

## Methods

### Institutional antibiotic stewardship

The State University of New York (SUNY) Upstate University Hospital is a 752-bed, tertiary-care, academic medical center located in Syracuse, New York. The antibiotic stewardship program (ASP) was established in 2014 and is co-led by an infectious diseases (ID) physician and an ID pharmacist. The ASP subcommittee reports to the medical executive committee within the health system and has representation from ID physicians (adult and pediatric), ID pharmacists (adult and pediatric), emergency medicine physicians, microbiology, and infection control. In 2018, the ASP was recognized as a Center of Excellence by the Infectious Diseases Society of America (IDSA).^17^

The ID physician has 0.25 full-time equivalents for antibiotic stewardship. The ID pharmacist has 1.0 FTE for antibiotic stewardship with the support of a pharmacy school-funded ID faculty member and a postgraduate year 2 ID pharmacy resident. The antibiotic stewardship service is staffed by ID pharmacist(s) with ID physician support and input weekdays from 08:00 a.m. to16:30 p.m., excluding weekends and holidays. Daily antibiotic stewardship activities include, but are not limited to, prospective audit and feedback, antibiotic preauthorization, discharge antibiotic prescription review, pharmacokinetic monitoring, renal dose adjustments, intravenous-to-oral conversions for highly bioavailable antibiotics, and drug information. Communication with the primary services occurs mostly through a chat function within the EMR. Chart notes from an ID physician and/or ID pharmacist can also be used as needed.

### AOS implementation

**Figure 1.**
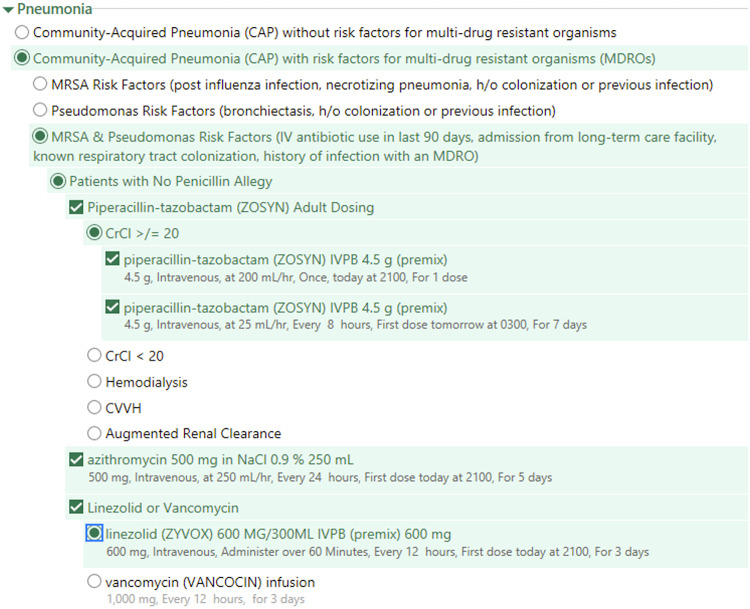
Example of community-acquired pneumonia empiric antibiotic order set embedded within the electronic medical record. Note. CVVH, continuous venovenous hemofiltration; CrCl, creatinine clearance; MDRO, multidrug resistant organism; MRSA, methicillin-resistant *Staphylococcus aureus*.

Prior to the development of the AOS, no clinical pathways or institutional ID management guidelines existed at our institution. In September 2018, our ASP implemented empiric AOS for pneumonia (community-acquired and hospital-acquired/ventilator-associated), urinary tract infection (UTI), intra-abdominal or gastrointestinal infection, skin and soft-tissue infection, bone and joint infection, meningitis and encephalitis, neutropenic fever, endovascular infection, pelvic infection, and oral cavity or neck infection. Antibiotic selection was based on guidance from IDSA when available, our antibiogram, and hospital formulary. All AOSs were directly incorporated into Epic software (Epic, Verona, WI). To increase visibility and awareness, AOSs were accessible by entering numerous keywords including any antibiotic order (eg, piperacillin-tazobactam) or by keying the word ‘antibiotic’ into the order queue, which would create a ‘pop-up’ for the AOS to be selected. Clinicians then selected the infection that they were managing, navigated through subheadings within the infection type, and applied patient-specific factors as appropriate. The AOS included drug names, dose, route, frequency, and suggested duration (ie, not limited to a single dose). Durations could later be truncated or extended as appropriate by the treating clinician. The AOS also incorporated patient-specific factors as appropriate. For example, the community-acquired pneumonia (CAP) AOS is subcategorized into those with or without risk factors for multidrug-resistant bacteria, alternative antibiotics for patients with a severe penicillin allergy, and dose adjustments for patients with renal dysfunction (Fig. 1). Antibiotic therapy could subsequently be de-escalated and/or adjusted as needed based on the patient’s clinical response and evolving microbiology and ID data.

Education regarding the AOS was provided during the initial implementation phase via pharmacy and therapeutics committee distribution, electronic newsletters to clinical staff, and educational seminars (eg, grand rounds, trainee physician orientation, antibiotic stewardship presentations). Trainee physicians were educated on the purpose, logistics, and use of the AOS annually in July by 2 investigators (S.J.T. and W.D.K.).

### Study outcomes

The primary outcome was to evaluate the impact of empiric AOS on the SAAR. Secondary outcomes included the following: antibiotic DOT per 1,000 PD; targeted use of fluoroquinolones, carbapenems, clindamycin, and vancomycin; AOS utilization; CDI cases; and clinicians’ perceptions of the AOS.

### Study design

This 5-year, quasi-experimental study comprised 5 phases. For each phase, data were collected over a 10-month period from August 1 to May 31, excluding June and July to minimize the influence of trainee physician cycling. Phase 1 spanned August 1, 2017, through May 31, 2018; phase 2 spanned August 2018 through May 31, 2019; phase 3 spanned August 2019 through May 31, 2020; phase 4 spanned August 2020 through May 31, 2021, and phase 5 spanned August 2021 through May 31, 2022. Phase 1 was before the AOSs were implemented (ie, before the intervention or control cohort). Phase 2 was the intervention period when the AOSs were implemented (September 2018). Phases 3, 4, and 5 were the first, second, and third postintervention periods, respectively. Phases 3–5 were all affected by the coronavirus 2019 (COVID-19) pandemic; however, COVID-19 was declared a pandemic during phase 3.^18^ The ASP did not introduce any novel antimicrobial stewardship initiatives and/or practices aside from the AOS. ASP physician and pharmacist staff numbers as well as daily ASP activities were consistent throughout all phases to better evaluate the impact of the AOS.

Unique to phase 5, the admitting trainee and attending physicians, which were the same throughout the entire week, were contacted every Monday via EPIC chat to provide a reminder on how to access the AOS and to strongly encourage their use. This group was targeted because the investigators deemed them to be the most likely clinicians to use the AOS when admitting a patient. We evaluated this targeted educational initiative during phase 5 only. Data related to adult patients aged ≥18 years were included, whereas all pediatric-related data were excluded. This study was reviewed and deemed exempt from review by the Institutional Review Board at SUNY Upstate Medical University (IRB no. 1545645-1).

A 20-question survey was also developed to evaluate clinician’s perceptions of the AOS after phase 5 (ie, June 2022). This survey was distributed to clinicians at SUNY Upstate University Hospital who staffed primary inpatient admission services including attending physicians, trainee physicians, and advanced practice providers (n = 247). Clinicians of consulting services were excluded from the survey because they do not actually place antibiotic orders but rather provide recommendations in their chart notes for antibiotic recommendations for the primary services to order. Incomplete survey responses were also excluded. A survey invitation letter with appropriate informed consent information was attached to the survey instrument and distributed electronically via email. By entering the survey, the respondent agreed to participate.

A Research Electronic Data Capture (REDCap) platform was used to design and collect survey responses. Survey questions were developed using the expert opinion of the ASP leadership of ID physicians and ID pharmacists. Participation in the survey was voluntary, but participation was encouraged. The survey was left open for 6 weeks with weekly reminder emails to nonresponders.

### Definitions

The SAAR and antibiotic DOT per 1,000 PD metrics were calculated using our institution’s data reported to the National Healthcare Safety Network. Targeted antibiotic orders were those that our ASP identified as ‘broad spectrum’ or ‘high risk’ based on their adverse effect profile. These included fluoroquinolones (intravenous or oral formulations of ciprofloxacin, levofloxacin, and moxifloxacin), carbapenems (meropenem, ertapenem, and imipenem-cilastatin), clindamycin (intravenous and oral formulations), and vancomycin (intravenous formulation). Targeted antibiotic orders were extracted from EPIC software during each period to represent total use. AOS utilization data were extracted from EPIC as the total signed antibiotic orders from the adult AOS order panel during phases 2–5. A CDI case was defined as a positive polymerase chain reaction (PCR) assay with positive enzyme immunoassay (EIA) as reported by our institution to potentially minimize identification of patients who may have colonization rather than have infection (ie, PCR positive, EIA negative) despite the lower sensitivity of the EIA. Notably, our institution converted to this PCR/EIA algorithm in February 2019, during phase 2 of our study. For the survey, clinicians included attending physicians, trainee physicians, and advanced practice providers.

### Statistical analysis

All statistical analyses including descriptive statistics were performed using R software (R Foundation for Statistical Computing, Vienna, Austria). Locally estimated scatterplot smoothing (LOESS) curves were utilized to display trend variables of interest between different periods (ie, phases 1–5) in an exploratory manner. To test the difference between periods, linear regression models were fitted using the generalized least squares (GLS) method. The ARMA (autoregressive moving average) correlation structure of order (*p*, *q*) was applied to model the corrections between monthly observations within each period. The Akaike Information Criterion (AIC) was used to select the appropriate values of *p* and *q*. In the model, period was considered the discrete variable. The overall significance of period was tested using the likelihood ratio test, and the comparisons of each period against the first were tested using the Wald test. A 2-tailed significance of *P* < .05 was considered statistically significant. For the survey analysis, continuous data were presented using median and interquartile range (IQR) whereas categorical data were presented using number and percentage. Comparisons of categorical data were performed using the χ^2^ test or the Fisher exact test, as appropriate. Comparisons of continuous data were performed using the Student *t* test or ANOVA, as appropriate.

## Results

**Figure 2.**
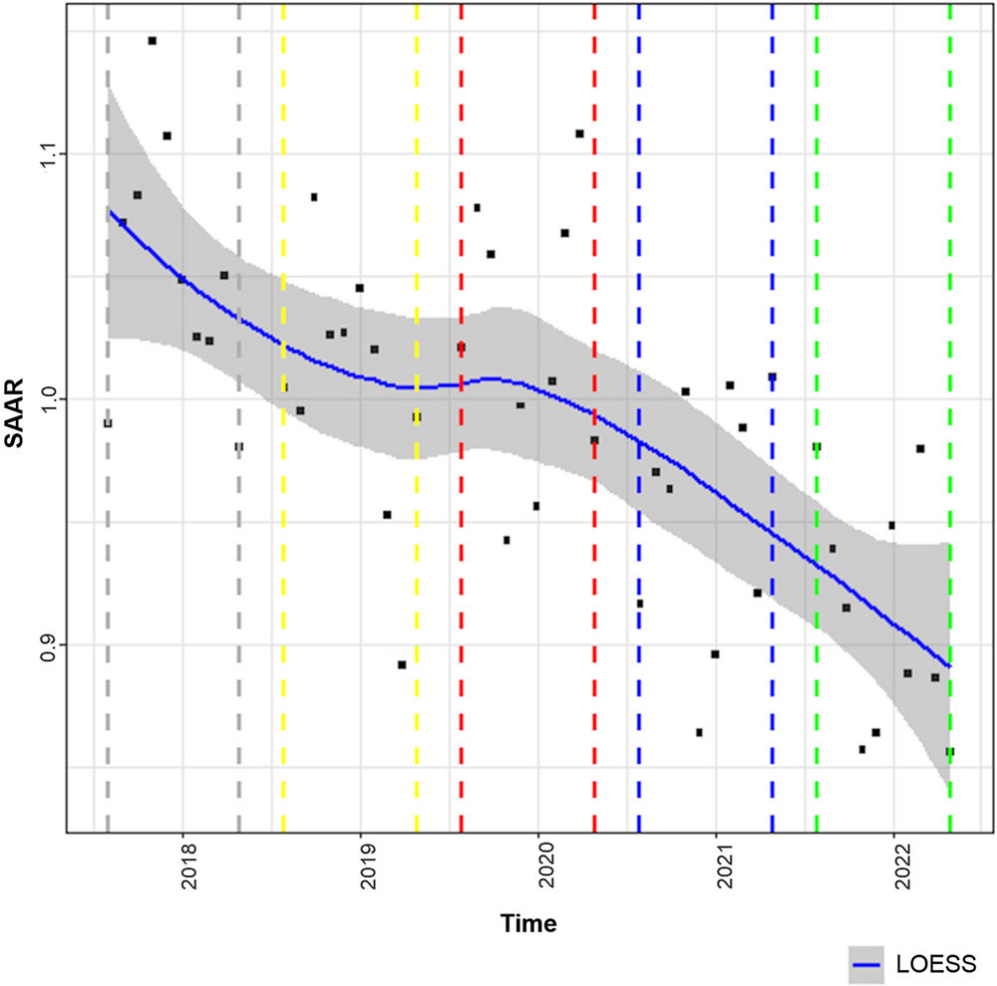
Standardized antimicrobial administration ratio evaluation. Solid blue line with grey shading is the LOESS curve. Dashed grey line indicates phase 1 (August 1, 2017–May 31, 2018). Dashed yellow line indicates phase 2 (August 1, 2018–May 31, 2019). Dashed red line indicates phase 3 (August 1, 2019–May 31, 2020). Dashed blue line indicates phase 4 (August 1, 2020–May 31, 2021). Dashed green line indicates phase 5 (August 1, 2021–May 31, 2022). Note. GLS, generalized least squares; LOESS, locally estimated scatterplot smoothing; SAAR, standardized antimicrobial administration ratio.

**Figure 3.**
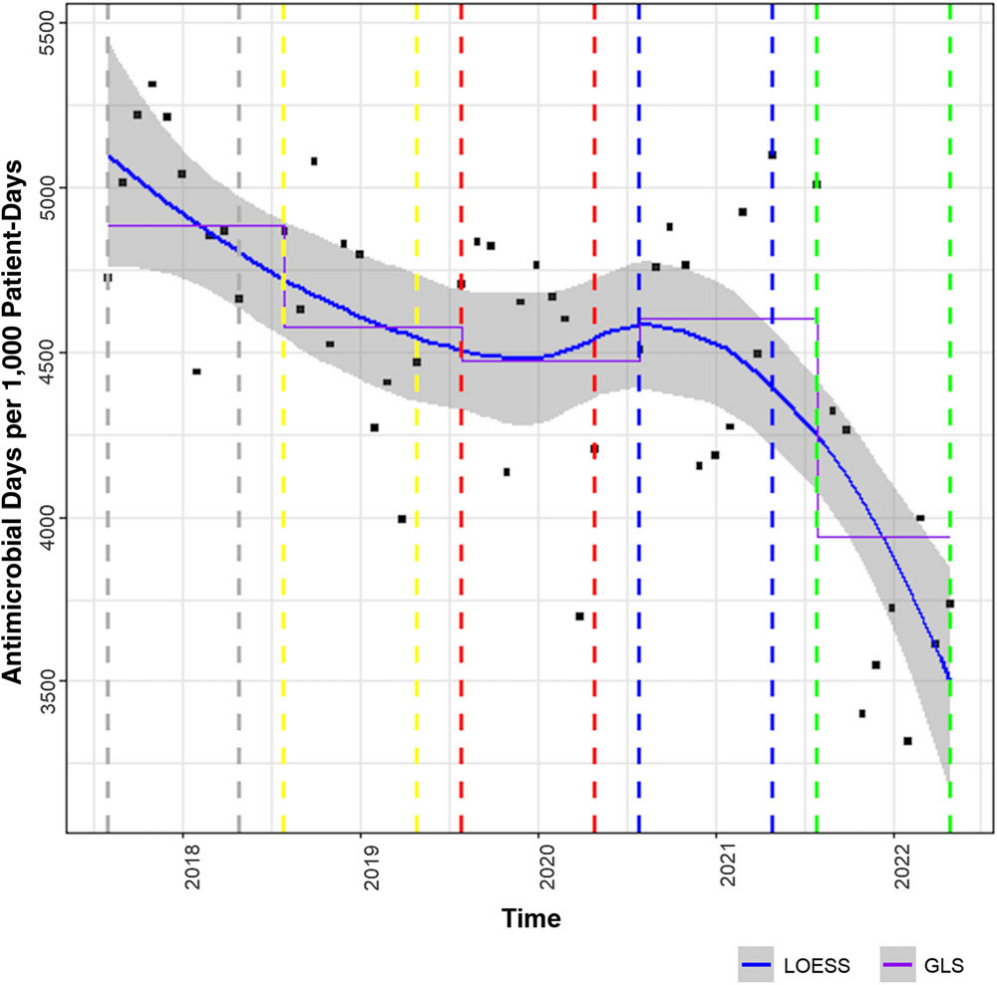
Antibiotic days of therapy per 1,000 patient days evaluation. Solid blue line with grey shading is the LOESS curve. Dashed grey line indicates phase 1 (August 1, 2017–May 31, 2018). Dashed yellow line indicates phase 2 (August 1, 2018–May 31, 2019). Dashed red line indicates phase 3 (August 1, 2019–May 31, 2020). Dashed blue line indicates phase 4 (August 1, 2020–May 31, 2021). Dashed green line indicates phase 5 (August 1, 2021–May 31, 2022). Note. GLS, generalized least squares; LOESS, locally estimated scatterplot smoothing.

**Figure 4.**
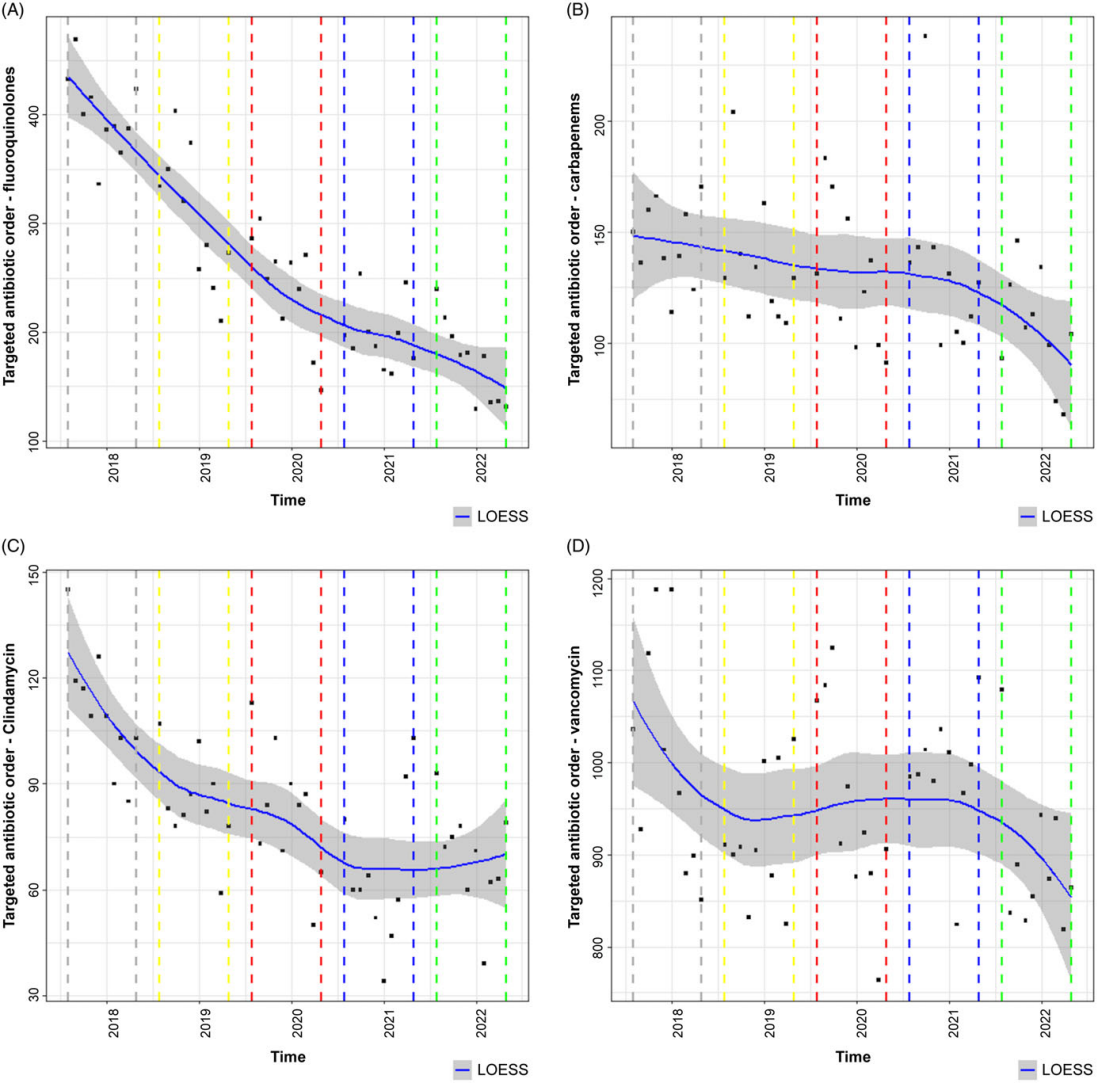
Targeted antibiotic order evaluation. (A) Fluoroquinolones. (B) Carbapenems. (C) Clindamycin. (D) Vancomycin. Solid blue line with grey shading is the LOESS curve. Dashed grey line indicates phase 1 (August 1, 2017–May 31, 2018). Dashed yellow line indicates phase 2 (August 1, 2018–May 31, 2019). Dashed red line indicates phase 3 (August 1, 2019–May 31, 2020). Dashed blue line indicates phase 4 (August 1, 2020–May 31, 2021). Dashed green line indicates phase 5 (August 1, 2021–May 31, 2022). Note. GLS, generalized least squares; LOESS, locally estimated scatterplot smoothing.

**Figure 5.**
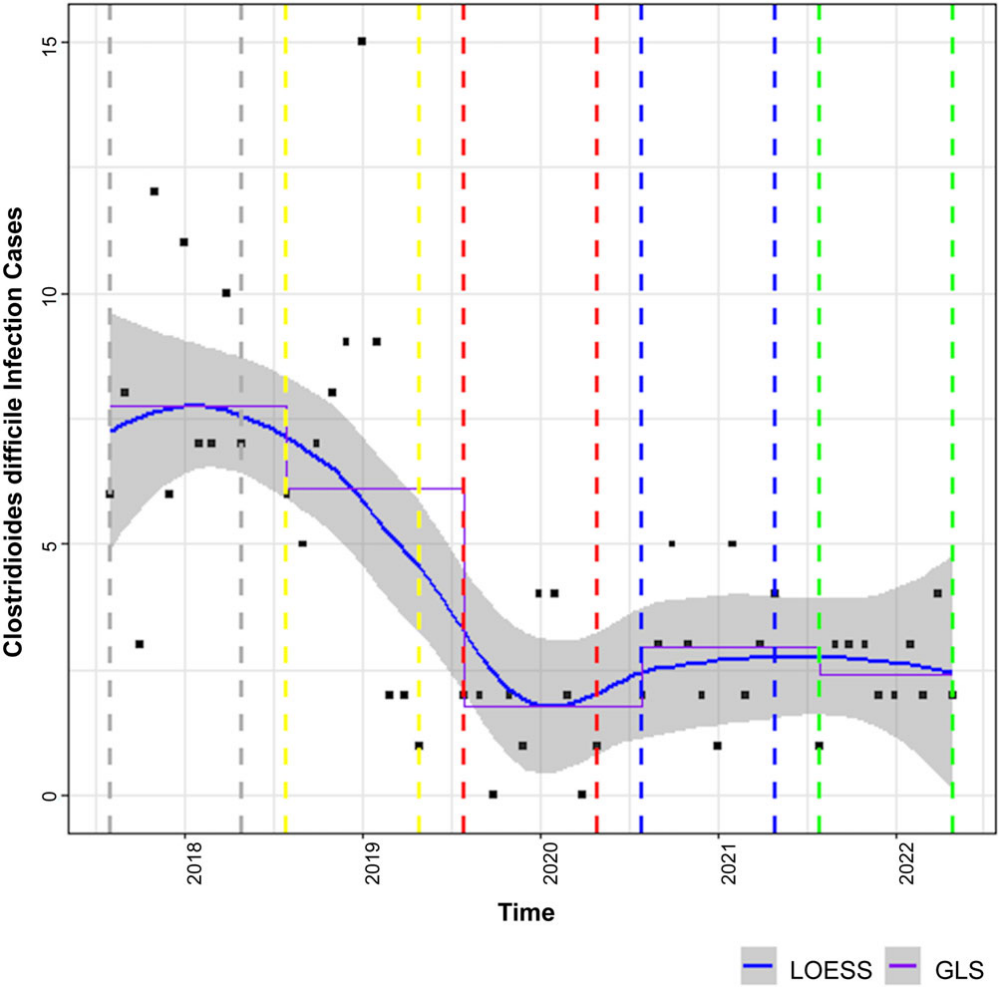
*Clostridioides difficile* infection cases. Solid blue line with grey shading is the LOESS curve. Dashed grey line indicates phase 1 (August 1, 2017–May 31, 2018). Dashed yellow line indicates phase 2 (August 1, 2018–May 31, 2019). Dashed red line indicates phase 3 (August 1, 2019-May 31, 2020). Dashed blue line indicates phase 4 (August 1, 2020–May 31, 2021). Dashed green line indicates phase 5 (August 1, 2021–May 31, 2022). Note. GLS, generalized least squares; LOESS, locally estimated scatterplot smoothing.

The LOESS curves to describe study outcomes associated with the AOS during phases 1–5 are displayed in Figures 2–5. Figure 2 describes the primary outcome of the SAAR during phases 1–5. A statistically significant decrease in the SAAR was observed from phase 1 to phase 5: 1.0 (95% confidence interval [CI], 1.0–1.1) versus 0.90 (95% CI, 0.8–1.0; *P*< .001). This difference was largely driven by a reduction in the SAAR for broad-spectrum antibacterial agents primarily used for hospital-onset infections for patients on general medicine wards. Secondary outcomes of antibiotic DOT per 1,000 PD, targeted antibiotic orders, and CDI cases displayed in Figures 3, 4, and 5, respectively. From phase 1 to phase 5, a statistically significant decrease in the DOT per 1,000 PD was detected: 4,884 (95% CI, 4,491–5,277) versus 3,939 (95% CI, 3383–4,495; *P* = .001).

Figure 4A–D describes targeted antibiotic orders during phases 1–5 with fluoroquinolones, carbapenems, clindamycin, and vancomycin representing Figure 4A, 4B, 4C, and 4D, respectively. From phase 1 to phase 5 in Figure 4A, a statistically significant reduction in fluoroquinolone orders was observed: 407 (95% CI, 350–464) versus 175 (95% CI, 94–256; *P* < .001). In Figure 4B, a statistically significant reduction in carbapenem orders was observed from phase 1 to phase 5: 147 (95% CI, 122–172) versus 106 (95% CI, 71–141; *P* = .024). In Figure 4C, a statistically significant reduction in clindamycin orders was observed from phase 1 to phase 5: 113 (95% CI, 92–135 versus 73 (95% CI, 42–103; *P* = .01). In Figure 4D, no statistically significant change was detected in vancomycin orders from phase 1 to phase 5: 991 (95% CI, 889–1,093 versus 902 (95% CI, 759–1,046; *P* = .221). The total signed antibiotic orders from the AOS for phases 2 (implementation phase), 3, 4, and 5 were 6,203, 10,800, 10,467, and 10,026, respectively. Figure 5 displays CDI cases during phase 1 through phase 5 where a statistically significant decrease in CDI cases was observed: 7.8 (95% CI, 10.1) versus 2.4 (95% CI, 5.8; *P* = .002). As mentioned previously, there was a change in CDI case diagnosis in February 2019 with PCR/EIA implementation. When comparing phase 3 to phase 5, no statistically significant difference in CDI cases were detected: 1.8 (95% CI, 1.6–5.1) versus 2.4 (95% CI, 1–5.8; *P* > .05).

In total, 58 of 247 clinicians completed the survey, for a response rate of 23.5%. Table 1 displays clinicians’ demographics and aggregated survey responses regarding perceptions of the AOS. Most respondents were trainee physicians (51.7%), had a practice area of internal medicine (44.8%), and encountered ≤20 patients per week with an infection (75.9%). On a 1-to-5 Likert scale, the median agreement was 4 (agree) or 5 (strongly agree) for all confidence and perception statements regarding the AOS. Clinicians strongly agreed that they were familiar with the AOS, that they were aware of the infections included, that they were confident in AOS location, that the AOS were easy to use, that the AOS helped them select the appropriate antibiotics including dosing and duration, and that they were well-informed about them. Furthermore, medicine and intensive care physicians indicated they were more familiar with the AOSs [median, 5.0 (IQR, 5.0–5.0) versus median, 4.0 (IQR, 4.0–5.0); *P* < .001] and were more likely to encourage colleagues to use the AOSs [median, 5.0 (IQR, 4.0–5.0) vs median 4.0 (IQR, 3.0–5.0; *P* = .013)] compared to surgery, emergency medicine, and other types of physicians, respectively. Clinicians agreed that their utilization of the AOSs increased throughout the previous year and that they encouraged their colleagues to utilize them.

**Table 1.** Clinician Demographics and Survey Responses Regarding Perceptions of the Empiric Antibiotic Order Sets (AOSs) (N = 58)

Clinician type	No. (%)^a^
Trainee physician	30 (51.7)
Attending physician	18 (31.0)
Advanced practice provider	10 (17.2)
**Practice/specialty area**	
Internal medicine	26 (44.8)
General surgery/surgical ICU	14 (24.1)
Emergency medicine	6 (10.3)
Cardiac surgery	3 (5.2)
Hematology/Oncology	3 (5.2)
Neurology	3 (5.2)
Pulmonary/medical ICU	3 (5.2)
**No. of years in practice from terminal degree**	
<3	24 (41.4)
3–5	8 (13.8)
6–10	11 (19.0)
11–15	6 (10.3)
16–20	2 (3.5)
21–30	4 (6.9)
>30	3 (5.2)
**No. of patients encountered with an infection within a given week**	
≤10	19 (32.8)
11–20	25 (43.1)
21–30	7 (12.1)
31–40	2 (3.5)
41–50	1 (1.7)
>50	4 (6.9)
**Confidence in and perceptions of the AOS^b^**	
I am familiar with the AOS, median (IQR)	5 (1)
I am aware of the infections that are included on the AOS, median (IQR)	5 (1)
I am confident in finding how to order antibiotics off the AOS panel, median (IQR)	5 (1)
The AOS help me select the appropriate antibiotic, dosing regimen, and duration, median (IQR)	5 (1)
The AOS are easy to use, median (IQR)	5 (1)
I was well-informed that our institution has AOS, median (IQR)	5 (1)
My utilization of the AOS increased throughout the past academic year, median (IQR)	4 (2)
I have encouraged my colleagues to use the AOS, median (IQR)	4 (2)
**Antibiotic stewardship and antibiotic prescribing education^b^**	
I am aware that AOS are recommended as an antibiotic stewardship initiative in the CDC Core Elements of Hospital Antibiotic Stewardship, median (IQR)	4 (2)
I received adequate education/training to prescribe antibiotics appropriately, median (IQR)	4 (2)
I have a good understanding of antibiotic stewardship principles, median (IQR)	4 (1)
I am interested in receiving more education on antibiotic stewardship (eg, continuing education, grand rounds), median (IQR)	4 (1)
**Trainee physicians’ perceptions of weekly reminders for the AOS^b^**	
I received a reminder via EPIC chat to encourage use of AOS as an admitting trainee physician, median (IQR)	5 (0.5)
I found the reminders useful as an admitting trainee physician, median (IQR)	5 (0)
**Primary barrier to AOS use**	
I do not believe there are any barriers to using the AOS	26 (44.8)
Differential diagnosis includes more than one type of infection	8 (13.8)
Attending physician preference is often not in alignment with the AOS	8 (13.8)
Unfamiliar with the AOS	7 (12.1)
Uncertain infection type	7 (12.1)
Antibiotic options within the order set are not appropriate for my patients	1 (1.7)
The AOS are not user friendly	1 (1.7)

Note. CDC, Centers for Disease Control and Prevention; ICU, intensive care unit; IQR, interquartile range.

a Units unless otherwise specified.

b Based on a likert scale of 1–5: 1, strongly disagree; 2, disagree; 3, neither disagree or agree; 4, agree; 5, strong agree.

On a Likert scale of 1 to 5, the median agreement was 4 (agree) for statements regarding antimicrobial stewardship and antibiotic prescribing. Despite clinicians agreeing that they received adequate education or training to prescribe antibiotic appropriately and had a good understanding of antimicrobial stewardship. They also agreed that they were interested in receiving more antimicrobial stewardship education. Trainee physicians strongly agreed that they were aware of receiving a reminder via EPIC chat to encourage AOS use and found these reminders useful when admitting patients. Most clinicians (44.8%) indicated that they did not perceive any identifiable barriers to using the AOS. Of the potential barriers listed, the primary barriers selected by respondents were the differential diagnosis including >1 type of infection (13.8%) or attending physician preference is often not in alignment with the AOS (13.8%).

## Discussion

To our knowledge, this is the largest, quasi-experimental study to evaluate the impact of AOS on antibiotic use metrics among hospitalized, adult patients. Implementation of AOS was associated with a statistically significant reduction in the SAAR and antibiotic DOT per 1,000 PD from the preimplementation period (phase 1) to 3 years after implementation (phase 5). We identified a nearly 50% reduction in the SAAR for broad-spectrum antibacterial agents predominately used for hospital-onset infections for patients on general medicine wards. This finding was not unexpected because the AOS for community-acquired infections (eg, CAP) steered clinicians away from broad-spectrum agents like cefepime and piperacillin-tazobactam in favor of ceftriaxone. There were also a statistically significant reductions in targeted antibiotics including fluoroquinolones, carbapenems, and clindamycin from the preimplementation period (phase 1) to 3 years after implementation (phase 5), but this reduction was not observed with vancomycin. It is unclear why there was no significant difference with vancomycin when reductions were demonstrated with other targeted antibiotics. The most significant reduction in DOT per 1,000 PD was observed for fluoroquinolones, whereas lesser reductions were seen for carbapenems and clindamycin. In addition to reductions in targeted antibiotic use, particularly with high-risk CDI antibiotics (eg, clindamycin, fluoroquinolones, carbapenems), a statistically significant difference in CDI cases was reported. However, this finding may be attributed to the change in our CDI testing algorithm. AOS utilization was generally consistent throughout phases 3–5, demonstrating that sustained usage supporting the changes in antibiotic use metrics were associated with the AOSs. Regarding clinician perceptions of the AOS, clinicians indicated that they were overall familiar with and found the AOS useful. Most indicated no perceived barriers to use.

Previous studies have evaluated AOS, but these have generally been limited to emergency departments, outpatient settings, or sepsis-specific protocols.^7–16,19–24^ Few studies describe interventions for multiple infections among adult, hospitalized patients. Seitz et al^25^ implemented AOSs for cystitis, pyelonephritis, cellulitis, and chronic obstructive pulmonary disease based on clinical practice guidelines, their antibiogram, and desire to avoid antibiotics with higher CDI risk. They demonstrated improved antibiotic selection, first-dose timing, and prescription duration with nearly all providers indicating the orders sets were easy to use.^25^ However, these AOSs were limited to the emergency department and represented fewer infections than in our study. Colmerauer et al^26^ evaluated AOS implementation on broad-spectrum antibiotic DOT per 1,000 PD for CAP. The AOSs were associated with a statistically significant reduction in median broad-spectrum antibiotic DOT per 1,000 PD (2 days vs 0 days; *P* < .001). However, this was limited to only one infection, and the study was conducted over 2 months in pre- and postintervention periods.^26^ Chan et al^27^ evaluated EMR-embedded AOS for cellulitis, UTI, and CAP. They reported statistically significant reductions in ciprofloxacin (mean, 16.6 DOT per 1,000 PD vs 13.6 DOT per 1,000 PD; *P* = .026) and moxifloxacin usage (mean, 9.3 DOT per 1,000 PD vs 5.2 DOT per 1,000 PD) during the study, which were consistent with the statistically significant reduction in fluoroquinolone use that we reported.^27^

Demonstrations of the impact of AOS on antimicrobial stewardship metrics are needed. Our findings indicate that AOSs can indeed reduce antibiotic consumption. As such, our experience and data provide a model for other institutions depending on their resources and analysis availability. Our study had several strengths. First, the study period was over 5 years with a 3-year postimplementation period. Thus, we evaluated not only the initial impact but also the sustainability of this AOS intervention. Second, we evaluated specific antibiotic use metrics such as SAAR and antibiotic DOT per 1,000 PD, which are important for ASP monitoring and reporting. Both demonstrated statistically significant reductions. We also evaluated the impact of an order set that encompassed multiple infection types rather than one with a singular infection type.

This study had several limitations. First, this study was performed at a single center with a specific AOS design. Our AOS evaluation was limited to hospitalized adult patients; thus, results may not be generalizable to other institutions, the outpatient setting, or pediatric patients. Second, although we reported a reduction in several antibiotic use metrics, unknown confounding variables could have affected these changes outside the AOSs. However, the ASP did not introduce any novel antimicrobial stewardship initiatives and/or practices, and no changes in ASP staffing models occurred throughout the postintervention period. Third, although we included the AOS utilization numerical data, we did not directly measure AOS usage relative to the total number of antibiotic orders because changes to the orders in the form of dose adjustments, renewals, and intravenous to oral conversion could potentially have undermined the results. Furthermore, providers could have also ordered the appropriate antibiotic regimen correctly through education and awareness of the AOS, even if it was not directly ordered from the AOS; thus, the AOS utilization uptake could potentially appear falsely lower. Lastly, the survey responses could be at risk for response bias by clinicians as well as nonresponse bias because the response rate was relatively low.

In conclusion, our findings have demonstrated that implementing AOS into the EMR as an ASP initiative was associated with a statistically significant reduction in SAAR, antibiotic DOT per 1,000 PD, selected antibiotic orders (ie, fluoroquinolones, carbapenems, and clindamycin), and CDI cases among hospitalized adult patients. Clinicians indicated that the AOSs were easy to use overall and that they helped them select the appropriate antibiotics.

## References

[1] Barlam TF , Cosgrove SE , Abbo LM , et al. Implementing an antibiotic stewardship program: guidelines by the Infectious Diseases Society of America and the Society for Healthcare Epidemiology of America. Clin Infect Dis 2016;62:e51–e77. 27080992 10.1093/cid/ciw118 PMC5006285

[2] Fleming-Dutra KE , Hersh AL , Shapiro DJ , et al. Prevalence of inappropriate antibiotic prescriptions among US ambulatory care visits, 2010–2011. JAMA 2016;315:1864–1873. 27139059 10.1001/jama.2016.4151

[3] Goff DA. Antibiotic stewardship: the health of the world depends on it. Hosp Pharm 2018;53:214–216. 30038435 10.1177/0018578718769964 PMC6050875

[4] Spellberg B , Guidos R , Gilbert D , et al. The epidemic of antibiotic-resistant infections: a call to action for the medical community from the Infectious Diseases Society of America. Clin Infect Dis 2008;46:155–164. 18171244 10.1086/524891

[5] Antibiotic prescribing and use. Centers for Disease Control and Prevention website. https://www.cdc.gov/antibiotic-use/stewardship-report/hospital.html. Published 2017. Accessed May 1, 2023.

[6] Goff DA , Kullar R , Bauer KA , File TM Jr. Eight habits of highly effective antimicrobial stewardship programs to meet the Joint Commission standards for hospitals. Clin Infect Dis 2017;64:1134–1139. 28203781 10.1093/cid/cix065

[7] Hauck LD , Adler LM , Mulla ZD. Clinical pathway care improves outcomes among patients hospitalized for community-acquired pneumonia. Ann Epidemiol 2004;14:669–675. 15380798 10.1016/j.annepidem.2004.01.003

[8] Worrall CL , Anger BP , Simpson KN , Leon SM. Impact of a hospital-acquired/ventilator-associated/healthcare-associated pneumonia practice guideline on outcomes in surgical trauma patients. J Trauma 2010;68:382–386. 19935109 10.1097/TA.0b013e318197bc74

[9] Ibrahim EH , Ward S , Sherman G , Schaiff R , Fraser VJ , Kollef MH. Experience with a clinical guideline for the treatment of ventilator-associated pneumonia. Crit Care Med 2001;29:1109–1115. 11395584 10.1097/00003246-200106000-00003

[10] Jenkins TC , Knepper BC , Sabel AL , et al. Decreased antibiotic utilization after implementation of a guideline for inpatient cellulitis and cutaneous abscess. Arch Intern Med 2011;171:1072–1079. 21357799 10.1001/archinternmed.2011.29

[11] Fine MJ , Stone RA , Lave JR , et al. Implementation of an evidence-based guideline to reduce duration of intravenous antibiotic therapy and length of stay for patients hospitalized with community-acquired pneumonia: a randomized controlled trial. Am J Med 2003;115:343–351. 14553868 10.1016/s0002-9343(03)00395-4

[12] Dellit TH , Chan JD , Skerrett SJ , Nathens AB. Development of a guideline for the management of ventilator-associated pneumonia based on local microbiologic findings and impact of the guideline on antimicrobial use practices. Infect Control Hosp Epidemiol 2008;29:525–533. 18510462 10.1086/588160

[13] Cook DA , Pencille LJ , Dupras DM , Linderbaum JA , Pankratz VS , Wilkinson JM. Practice variation and practice guidelines: attitudes of generalist and specialist physicians, nurse practitioners, and physician assistants. PLoS One 2018;13:e0191943. 29385203 10.1371/journal.pone.0191943 PMC5792011

[14] Fargo EL , D’Amico F , Pickering A , Fowler K , Campbell R , Baumgartner M. Impact of electronic physician order-set on antibiotic ordering time in septic patients in the emergency department. Appl Clin Inform 2018;9:869–874. 30517970 10.1055/s-0038-1676040 PMC6281440

[15] Wilde AM , Nailor MD , Nicolau DP , Kuti JL. Inappropriate antibiotic use due to decreased compliance with a ventilator-associated pneumonia computerized clinical pathway: implications for continuing education and prospective feedback. Pharmacotherapy 2012;32:755–763. 23307523 10.1002/j.1875-9114.2012.01161.x

[16] Vuong L , Kenney RM , Thomson JM , et al. Implementation of indication-based antibiotic order sentences improves antibiotic use in emergency departments. Am J Emerg Med 2023;69:5–10. 37027958 10.1016/j.ajem.2023.03.048

[17] IDSA Antimicrobial Stewardship Centers of Excellence. Infectious Diseases Society of America website. https://www.idsociety.org/clinical-practice/antimicrobial-stewardship/centers-of-excellence/. Published 2023. Accessed April 3, 2023.

[18] Coronavirus disease (COVID-19) pandemic, 2023. World Health Organization website. https://www.who.int/europe/emergencies/situations/covid-19. Accessed April 11, 2023.

[19] Nichols KR , Petschke AL , Webber EC , Knoderer CA. Comparison of antibiotic dosing before and after implementation of an electronic order set. Appl Clin Inform 2019;10:229–236. 30943571 10.1055/s-0039-1683877 PMC6447403

[20] Braxton CC , Gerstenberger PA , Cox GG. Improving antibiotic stewardship: order set implementation to improve prophylactic antimicrobial prescribing in the outpatient surgical setting. J Ambul Care Manag 2010;33:131–140. 10.1097/JAC.0b013e3181d91680 20228636

[21] Blow C , Harris J , Murphy M , et al. Evaluation of a pharmacist-led antibiotic stewardship program and implementation of prescribing order sets. J Am Pharm Assoc 2021;61 suppl 4:S140–s146. 10.1016/j.japh.2021.01.030 33642241

[22] Hiensch R , Poeran J , Saunders-Hao P , et al. Impact of an electronic sepsis initiative on antibiotic use and health care facility-onset *Clostridium difficile* infection rates. Am J Infect Control 2017;45:1091–1100. 28602274 10.1016/j.ajic.2017.04.005

[23] Zahlanie Y , Mang NS , Lin K , Hynan LS , Prokesch BC. Improved antibiotic prescribing practices for respiratory infections through use of computerized order sets and educational sessions in pediatric clinics. Open Forum Infect Dis 2021;8:ofaa601. 33553470 10.1093/ofid/ofaa601 PMC7849952

[24] Buehrle DJ , Shively NR , Wagener MM , Clancy CJ , Decker BK. Sustained reductions in overall and unnecessary antibiotic prescribing at primary care clinics in a Veterans’ Affairs healthcare system following a multifaceted stewardship intervention. Clin Infect Dis 2020;71:e316–e322. 31813965 10.1093/cid/ciz1180

[25] Seitz RM , Wiley Z , Francois CF , Moran TP , Rupp JD , Sexton ME. Improved empiric antibiotic prescribing for common infectious disease diagnoses using order sets with built-in clinical decision support in the emergency department. Infect Control Hosp Epidemiol 2022;43:672–674. 33686931 10.1017/ice.2021.73

[26] Colmerauer JL , Linder KE , Dempsey CJ , Kuti JL , Nicolau DP , Bilinskaya A. Impact of order-set modifications and provider education following guideline updates on broad-spectrum antibiotic use in patients admitted with community-acquired pneumonia. Hosp Pharm 2022;57:496–503. 35898261 10.1177/00185787211055797 PMC9310309

[27] Chan A , Kapur A , Langford B , Downing M. 1046. Evaluating the outcomes of embedding antimicrobial stewardship order sets in the general medicine admission electronic order set: a retrospective study. Open Forum Infect Dis 2019;6 suppl 2:S368–S369.

